# Prosthetic Rehabilitation in a 12-Year-Old Visually Impaired Girl With Congenitally Missing Permanent Teeth Following Anterior Tooth Extraction: A Case Report

**DOI:** 10.7759/cureus.77752

**Published:** 2025-01-20

**Authors:** Minakshi Tripathy, Priyanka Singh, Tanvi Saraf, Ashwin Jawdekar, Shreyas Neelkanthan

**Affiliations:** 1 Department of Pediatric and Preventive Dentistry, Bharati Vidyapeeth (Deemed to be University) Dental College and Hospital, Navi Mumbai, IND

**Keywords:** flexible denture, maxillary anterior denture teeth, maxillary anterior teeth, prosthetic rehabilitation, removable partial dentures, rpd, visually impaired, visually impaired children

## Abstract

Dental health is integral to a child's overall physical and psychological development, especially during the formative years. Children with visual impairment face distinct challenges in maintaining oral hygiene and receiving dental care, often due to difficulty in accessing appropriate resources, understanding dental procedures, and coping with anxiety in unfamiliar clinical settings. Inclusive dentistry emphasizes the importance of addressing the unique oral health needs of individuals with disabilities, particularly children, to ensure equitable access to care. Visually impaired children often require specialized dental management strategies that prioritize their comfort while overcoming communication barriers. Techniques such as Tell-Feel-Do (TFD) and Audio-Tactile-Performance (ATP) have proven effective in fostering a positive dental experience and achieving comprehensive treatment outcomes.

This case report presents the case of a visually impaired girl who visited the outpatient Department of Pediatric and Preventive Dentistry with a chief complaint of pain in the upper front region of her jaw over the past month. She had no previous dental history. The case demonstrates the importance of meticulous planning and individualized care to successfully manage the dental needs of visually impaired children. It also highlights practical approaches to delivering effective and inclusive dental treatment.

## Introduction

Dental health is integral to a child's overall physical and psychological development, especially during formative years. Children with visual impairment face distinct challenges in maintaining oral hygiene and receiving dental care, often due to difficulty in accessing appropriate resources, understanding dental procedures, and coping with anxiety in unfamiliar clinical settings. The replacement of extracted anterior teeth in such children is particularly important, as these teeth play a crucial role in appearance, speech, and chewing, all of which contribute to social interaction and self-esteem [1]. For a visually impaired child, the absence of anterior teeth can significantly impact their confidence and ability to engage socially. Dental interventions must therefore go beyond clinical treatment and incorporate strategies sensitive to the child’s sensory limitations. Effective management requires an understanding of the unique needs of visually impaired patients, including clear communication through tactile, auditory, and descriptive methods. Behavioral guidance and anxiety management techniques are also essential to ensure cooperation and comfort during treatment [2]. 

Visually impaired children often face significant challenges in maintaining oral health, leading to a higher prevalence of dental issues compared to their sighted peers. Studies have reported that the prevalence of dental caries in visually impaired children ranges from 40% to 81.9% [3]. Additionally, the overall prevalence of dental caries has been found to be 60% in visually impaired children, compared to 31.5% in normal children [4]. These children also exhibit poorer oral hygiene. A study reported that 67% of visually impaired children had gingival inflammation, indicating a high prevalence of periodontal issues in this population [5]. Comprehensive dental management of children with sensory limitations requires a multidisciplinary approach tailored to each child's unique needs. Guidelines have been referred to and followed. A thorough evaluation was done to understand the child's sensory sensitivities, communication abilities, and medical history. This assessment informs the development of a personalized care plan [6]. By integrating these protocols, dental professionals can effectively manage the oral health of children with sensory limitations, promoting better health outcomes and enhancing the quality of life (QoL) for these individuals.

These findings underscore the need for specialized dental care and targeted preventive strategies to address the unique oral health challenges faced by visually impaired children.

This case report details the comprehensive dental management of a 12-year-old girl with visual impairment who required the replacement of extracted anterior teeth. The approach involved meticulous planning to address the child's functional and aesthetic needs while accommodating her visual challenges.

## Case presentation

A 12-year-old female patient reported to the outpatient Department of Pediatric and Preventive Dentistry with a chief complaint of pain in the upper front region of her jaw for a month, having a history of dull aching pain. The medical history revealed visual impairment since birth. According to the parents, the patient was delivered at full term by normal delivery. On general examination, the patient was well-oriented and well-built. Extraoral examination revealed the face was asymmetrical without any obvious deformity. Bilaterally submandibular lymph nodes were palpable and non-tender.

A complete history was taken, and an informed consent was signed by the parent before the start of the treatment.

Slow, clear, and concise instructions were given. The child was informed before moving from one place to another and accompanied by a postgraduate student to the dental chair and was made comfortable in the dental chair. The dental chair movements (like up, down, etc.) were verbally first explained to the patient, and the chair movements were done. The child was instructed and assisted to place her hands on the side handrest of the dental chair, and due care was taken to avoid any sudden jerks. Her attention was gained by lightly patting her shoulder and hands.

The Frankl Behavior Rating (FBR) scale was used to grade the child's behavior. The FBR of the patient was positive (+). The Caries Risk Assessment for Treatment (CRAFT) was conducted for the patient, and based on the findings, she was classified as being at high risk for dental caries. As a result, she was advised to reduce her sugar intake, and a weekly diet chart was created to help monitor her nutritional habits. The patient and her family were given specific guidelines on how to limit sugar consumption, and the family was educated about the hidden sources of sugar in everyday food items and how to make healthier food choices. A diet chart was designed that included balanced meal suggestions, emphasizing low-sugar and nutritious foods. The chart was created in a format accessible for the family to easily follow and track. Parents or caregivers play a crucial role in helping the child follow the dietary guidelines. Since the patient was visually impaired, family members were responsible for assisting with meal preparation and portion control, ensuring adherence to the prescribed diet. The support system created by the family helped ensure that the child followed through with the dietary changes effectively.

The impact of diet on oral and general health was explained, and caries-related preventive counseling was done for the parent and child. The importance of good oral health and proper brushing methods was explained using the Audio-Tactile-Performance (ATP) technique. 

**Figure 1:**
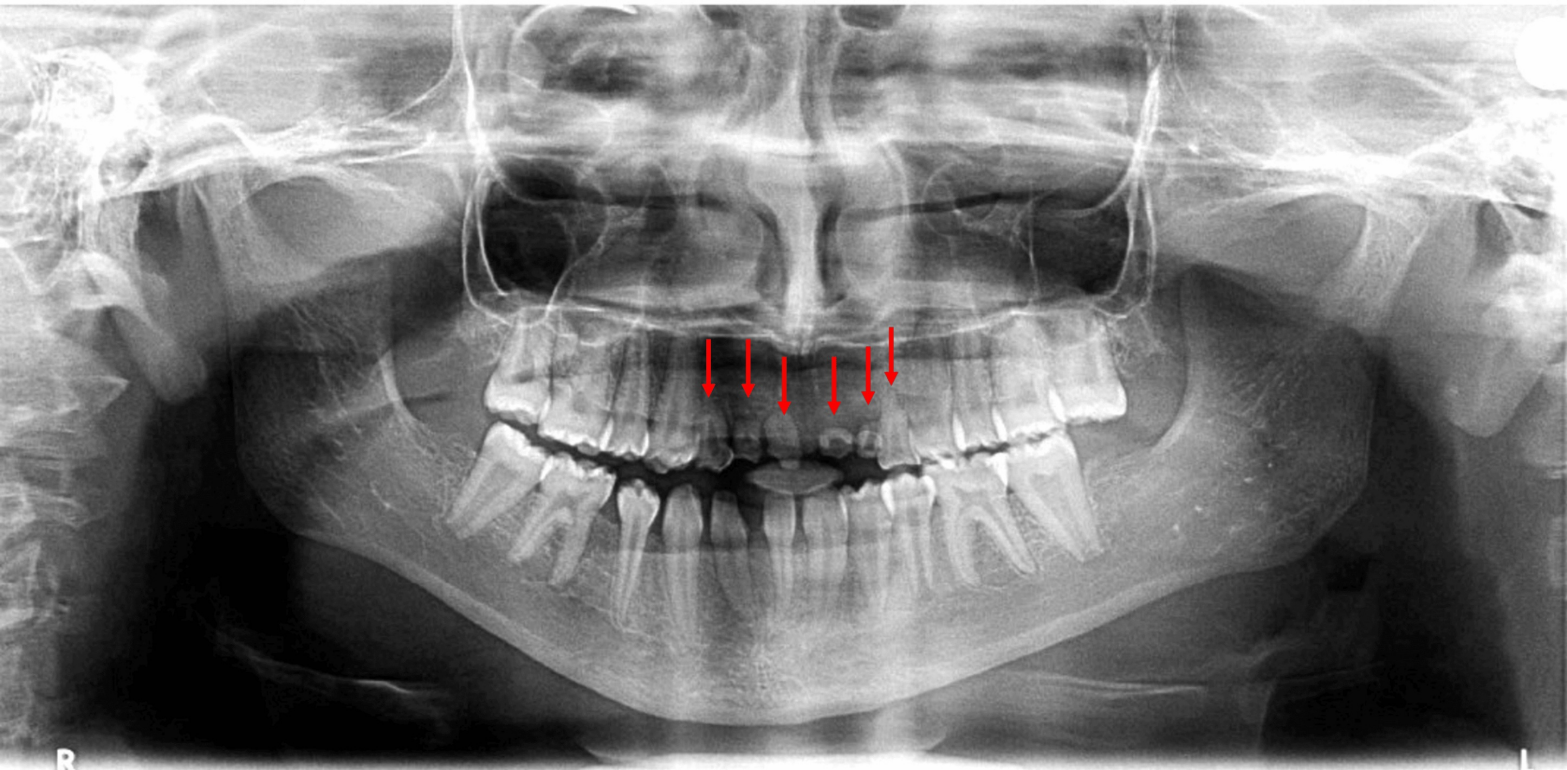
Preoperative orthopantomogram Root resorption with retained deciduous 51, 52, 53, 61, 62, and 63 and the absence of permanent tooth buds of 11, 12, 13, 21, 22, 23, 41, 45, and 32 are noted.

On dental examination, root pieces were present with 51, 52, 61, and 62; smooth surface caries involving enamel, dentin, and pulp 53, 63; and occlusal caries involving enamel 36, 46. The orthopantomogram (OPG) revealed root resorption with retained deciduous 51, 52, 53, 61, 62, and 63 and the absence of permanent tooth buds of 11, 12, 13, 21, 22, 23, 41, 45, and 32 (Figure 1). Conical-shaped teeth present with 31, 42. Investigations like complete blood count (CBC), bleeding time (BT), clotting time (CT), and OPG were advised. All the reports were in the normal range.

The behavior management technique employed here was the Tell-Feel-Do (TFD) technique before introducing the dental treatment. Dental instruments like a mouth mirror, air/water syringe, and handpiece (airotor) were explained to the child (Tell), and following this, she was allowed to feel them with her hands (Feel). The procedure was done in the exact same sequence as it was explained to the child without any deviation (Do). The examinations and other dental procedures were conducted using this approach only, except for the use of sharp instruments like needles, dental probes, etc.

The visit-wise protocol was followed for the treatment (Table 1).

**Table 1:** Outline of the treatment summary with visit-wise protocol OPD: outpatient department; RMGIC: resin-modified glass ionomer cement; w.r.t: with respect to; RPD: removable partial denture

Treatment needed	Preventive measure	Visit-wise protocol
Extraction w.r.t. 51, 52, 53, 61, 62, and 63	Office care measures: Oral prophylaxis, topical fluoride gel application	Visit 1: OPD consultation and behavior management
Restoration w.r.t. 36, 46	Home care measures: Avoid sugary foods, increase intake of fruits and vegetables, brush twice daily with fluoridated toothpaste, and use fluoride mouthwash once a day.	Visit 2: Oral prophylaxis and topical fluoride gel application; Visit 3: RMGIC restoration w.r.t. 36; Visit 4: RMGIC restoration w.r.t. 46; Visit 5: Extraction w.r.t. 51, 52, 61, 62
Prosthetic rehabilitation		Visit 6: Extraction w.r.t. 53; Visit 7: Extraction w.r.t. 63; Visit 8: Impressions of maxillary and mandibular arches. Visit 9: Trial of waxed-up denture; Visit 10: Delivery of the RPD

A thorough oral examination and radiological investigation were done on the first day of the visit. Continous conversation was maintained in order to develop a good rapport with the child and to gain her confidence. All the modifications made her very comfortable and stress-free during the treatment.

Oral prophylaxis was followed by topical 1.23% acidulated phosphate fluoride (APF) gel application in her second appointment. The scaler tip was used on the child’s fingernails to make her feel the sound and vibrations. She was also made to touch and smell the fluoride gel before its application.

In the third and fourth appointments, resin-modified glass ionomer cement (RMGIC) restorations were done with respect to (w.r.t) caries in 36 and 46, respectively. Here, she was explained about the use of an airotor and tooth restoration. It began verbally describing the airotor and its purpose in simple, reassuring language. The child was then made to touch and explore the airotor and other instruments before starting the procedure and also demonstrated the sound using euphuisms like “motorcycle.” Finally, the airotor was placed near her tooth, allowing her to feel the vibration and hear the sound directly on her tooth along with continuous verbal reassurance.

In the fifth, sixth, and seventh appointments, extraction was done w.r.t 51, 52, 53, 61, 62, and 63 under local anesthesia using 2% lignocaine with 1:100,000 adrenaline using the infiltration technique. Before giving the injection, she was told that she was going to feel a tiny pinch on her gum that would kill the tooth bug, and we would be able to remove the bug without much pain.

**Figure 2:**
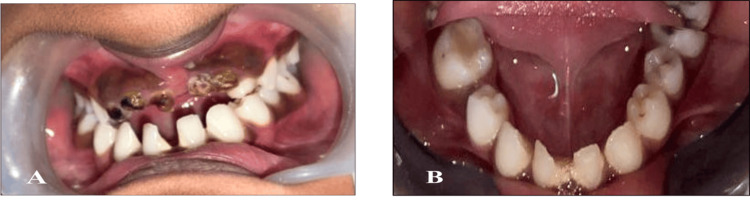
Preoperative clinical photograph A: At occlusion; B: Mandibular arch

**Figure 3:**
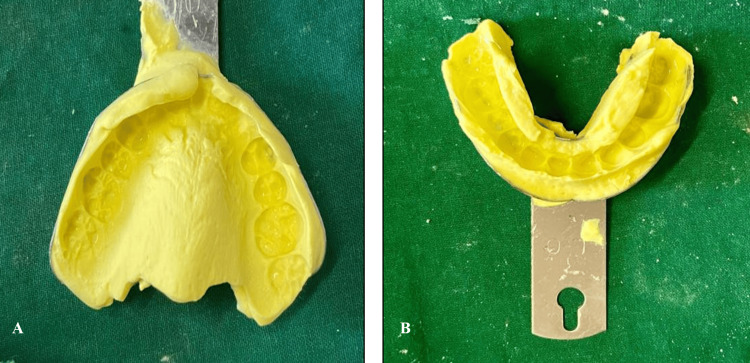
Primary impression of maxillary and mandibular arches using alginate impression material A: Maxillary arch; B: Mandibular arch

On the eighth appointment, a primary impression was recorded using irreversible hydrocolloid impression material (alginate) (Figure 2), and a master cast was poured using type III gypsum. Denture base fabrication using cold cure acrylic resin was done, and an occlusal rim was made. Teeth selection of appropriate shade was done. Required modification of teeth size and shape was done, and a trial denture was made (Figure 3). During the procedure, she was allowed to touch and feel the alginate paste. It was described to her as soft and squishy, like play dough, and would taste like fruity bubblegum. She was also made to touch the impression tray and feel its shapes and edges. Every single step was explained to her, from placing the impression tray, mixing the alginate material, removing the impression tray, etc. While the impression tray was inside her mouth, counting was done aloud to make her understand the duration.

The ninth appointment involved a try-in of the waxed-up denture, evaluation of esthetics, phonetics, and denture extensions. The tenth appointment was the delivery of the removable partial denture (RPD).

The parents were given postoperative instructions like removing and rinsing the denture after eating every meal, brushing the denture daily (only with the use of a soft bristle toothbrush), soaking the denture overnight in water, and asked to contact the dentist immediately if they experience a loose fit or ulcer/sore spot due to the denture. 

The patient returned for a follow-up visit after 24 hours, one week, four weeks, and six weeks, and necessary adjustments were made, and it was observed that both the patient and the parents had successfully adapted to the postoperative care instructions. Additionally, there was a noticeable improvement in the patient's psychosocial well-being. Based on the FBR, the patient's score improved from "positive (+)" to "definitely positive (++)". Training sessions for the insertion and removal of the denture with parents and the patient were done at each appointment. 

Follow-up for six months was done, emphasizing the importance of ongoing monitoring and maintenance. Also, definitive treatment, either implant or cast partial denture (CPD), will be done once the growth is complete. The success of the treatment plan extends beyond clinical outcomes to encompass the patient's overall well-being. Generally, for the visually impaired child, successful use of such a technique in dental situations is suggested by the authors. 

**Figure 4:**
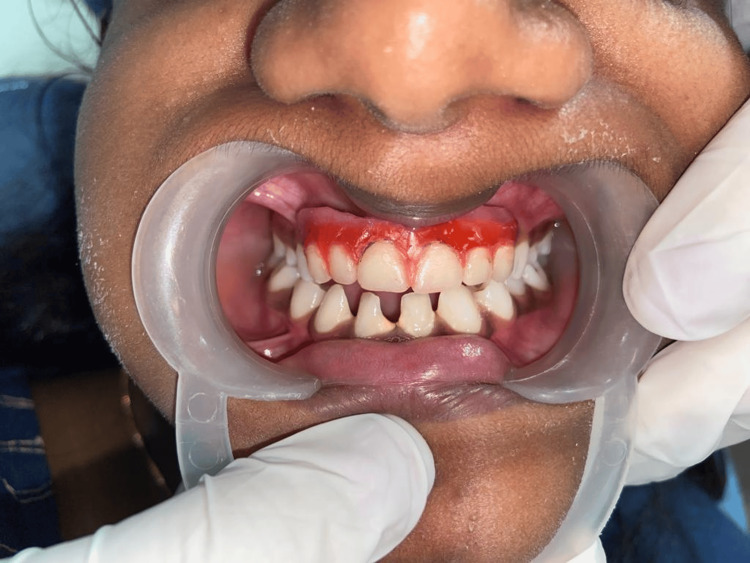
Postoperative intraoral view showing the restored teeth in occlusion.

**Figure 5:**
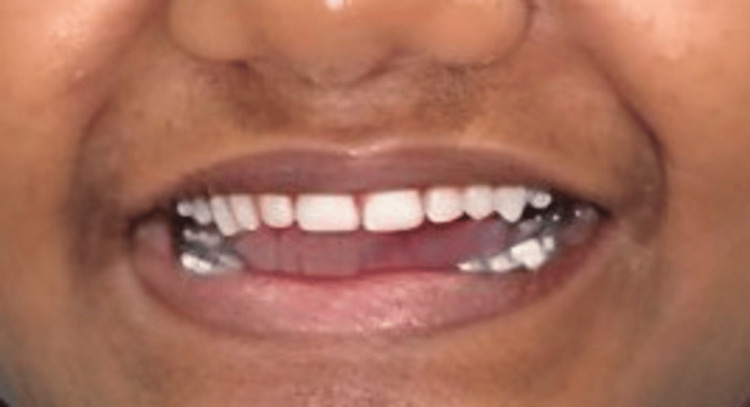
Postoperative profile of the child after the replacement of the teeth.

The patient and the parents were instructed to report to the department as soon as they noticed looseness, tightness, or any discomfort in the denture (due to constant growth). Follow-up was done for six months, a total of two times. The child reported satisfaction related to wearing and chewing. The parents expressed their pleasure related to her facial aesthetic. The outcome of the dental rehabilitation is shown (Figures 4, 5).

## Discussion

Visual impairment, a condition that profoundly impacts sensory perception and daily functionality, presents unique challenges and requires innovative adaptations [7]. According to the World Health Organization (WHO), low vision refers to a visual acuity ranging from 20/70 to 20/400 with the best correction possible or a visual field limited to 20 degrees or less. In contrast, blindness is characterized by a visual acuity worse than 20/400 with the best correction or a visual field of 10 degrees or less” [1]. Treatment strategies included patient-centered communication, behavioral techniques to reduce anxiety, and prosthetic rehabilitation to restore oral function and appearance. The successful outcome was achieved through a multidisciplinary approach, highlighting the collaboration between pediatric dentists, prosthodontists, and caregivers. This underscores the importance of individualized dental care plans for children with visual impairment, demonstrating how adaptive techniques can enhance patient comfort and treatment effectiveness. This case provides valuable insights into managing similar clinical situations, ultimately improving the child’s QoL and confidence.

This case report presents the preventive, restorative, and prosthetic management of a 12-year-old girl with visual impairment.

Research indicates that the prevalence of dental caries in children with visual impairment is comparable to that of their peers without visual impairment. However, as children with special healthcare needs (SHCNs) grow older, their oral health tends to decline more rapidly than that of the general population. A systematic review by Davis and Anders highlighted that individuals with SHCNs have a higher prevalence of untreated dental caries and periodontal disease compared to those in the general population [2].

Individuals with visual impairment often encounter challenges in maintaining optimal oral health. The extent of their impairment can affect their ability to detect issues like dental caries, gingival bleeding, or, more concerning, potentially suspicious lesions [8].

Effective communication and empathy are fundamental in managing pediatric patients with visual impairment. While numerous behavior management techniques exist in the literature for pediatric patients, visually impaired patients necessitate a modified and amalgamated approach tailored to individual needs and acceptance. These children have a lack of awareness about the importance of oral health care, challenges in expressing oral health concerns, and anxiety related to dental procedures; hence, methods like TFD and ATP were used in this instance to promote the child's active engagement [9].

The ATP technique was designed by Hebbal et al. in 2001 to educate visually impaired children regarding oral hygiene maintenance. It incorporates three key components: audio, tactile, and performance. Initially, the procedure is explained to the child through verbal instructions (audio component). A large-sized model is then used to help the child feel the teeth and understand the procedure (tactile component). The child is encouraged to feel their own teeth with their tongue to identify any hard deposits or irregularities and then brush their teeth under supervision (Performance). Empathetic communication is used throughout, motivating the child to practice oral hygiene independently [10].

A systematic review carried out by Kumaraguru et al. (2024), concludes that the ATP technique resulted in notable improvements in oral health compared to traditional methods [10].

The purpose of prosthetic rehabilitation (interim prosthesis) was to ease speech and mastication along with appearance, to develop normal temporomandibular joint (TMJ) function, to act as a space maintainer, and to avoid the development of harmful habits like tongue thrusting. Complete or partial removable dentures have been used successfully in numerous patients having oligodontia or hypodontia. In this case, flexible dentures have shown a novel approach towards the fulfillment of golden standards of prosthetic rehabilitation.

Dental management for patients with sensory impairments: Visually impaired patients face multiple challenges in dental care, including difficulty detecting dental issues and using oral hygiene aids, as well as reduced access to dental services due to transportation barriers and insufficient healthcare provider training. Hence, it is essential to consider the unique challenges faced by these individuals and plan the treatment accordingly [8, 9].

Additionally, visual impairment can impact oral health and contribute to psychological distress. Effective dental management for these patients requires tailored oral hygiene instruction using tactile tools and a strong emphasis on preventive care. Dentists play a crucial role in educating and supporting visually impaired patients to ensure their oral health and overall well-being [11].

Implementing tailored oral health education strategies that accommodate sensory limitations has been shown to significantly improve clinical outcomes in visually impaired children. A study reported a notable reduction in mean plaque scores over a six-month period. Specifically, the mean plaque score decreased from 1.24 ± 0.47 at baseline to 1.10 ± 0.17 after six months, indicating improved oral hygiene. Additionally, there was a shift in gingival health status, with the percentage of children exhibiting moderate gingivitis decreasing from 87.8% at baseline to 52.7% after six months, reflecting enhanced periodontal health [12]. These findings underscore the efficacy of customized educational interventions in promoting better oral health among visually impaired children. The long-term impact of such tailored strategies includes the potential for sustained oral health maintenance, reduced incidence of dental diseases, and an overall enhancement in QoL for visually impaired individuals.

This case report provides significant insights into the field of inclusive dentistry by addressing the unique challenges of treating a visually impaired child with congenital tooth agenesis following anterior tooth extraction. By focusing on the application of tailored prosthetic rehabilitation techniques, this case demonstrates how inclusive dental practices can be effectively implemented to meet the specific needs of patients with disabilities. The rehabilitation approach not only ensures the restoration of function and aesthetics but also emphasizes the importance of patient-centered care in pediatric dentistry. This case contributes to the advancement of inclusive dentistry by showcasing strategies that accommodate sensory impairments, such as the use of clear, accessible communication methods (e.g., ATP techniques), which are essential in achieving successful outcomes with children who have limited visual or other sensory input. Additionally, the case highlights the interdisciplinary approach required for comprehensive care, involving coordination between the dentist, parents, and any other healthcare professionals involved in the child's well-being.

This report also expands on how individualized treatment plans can address not only the dental needs of children with disabilities but also the broader implications for their overall health, including improved self-esteem and QoL.

This report reinforces the need for further research and training in inclusive dental practices, ultimately contributing to the development of more effective and accessible dental services for all children, regardless of their physical or sensory limitations.

## Conclusions

The successful rehabilitation of anterior teeth using a flexible removable partial denture highlights the importance of adaptive strategies customized to address individual challenges. Dental professionals can significantly improve the oral health outcomes and overall well-being of visually impaired patients. 

In conclusion, the study emphasizes the importance of raising awareness and providing education within the dental community to promote a patient-centric approach for individuals with disabilities. It highlights the crucial role of empathy and careful planning in delivering inclusive and comprehensive care, ensuring that all patients receive the attention and treatment they deserve.
